# Bridging research and practice for dementia care: strategies and challenges of public and private funders in the dissemination and implementation of dementia research

**DOI:** 10.1186/s12961-025-01440-7

**Published:** 2026-01-14

**Authors:** Eden Meng Zhu, Martina Buljac-Samardžić, Kees Ahaus, Robbert Huijsman

**Affiliations:** 1 Erasmus School of Health Policy and Management, PO Box 1738, 3000 DR Rotterdam, The Netherlands; 2 Centre for Behavioural and Implementation Science Interventions (BISI), Yong Loo Lin School of Medicine, National University of Singapore, Singapore, Singapore

**Keywords:** Integrated knowledge translation, Dementia, Implementation science, Capacity-building, Research funders

## Abstract

**Background:**

Although dementia research agendas increasingly prioritize dissemination and implementation (D&I) of research findings, there is still limited understanding of the role and activities of dementia research funders. Implementation science literature offers theories, frameworks and tools to integrate diverse stakeholder perspectives, supporting the translation of research evidence into practice and policy. This study identifies and categorizes the D&I strategies and related challenges, faced by public and private dementia research funders in the Netherlands. This study aims to provide evidence that clarifies the roles of public and private dementia research funders and offers guidance for planning and executing dementia research D&I. This study contributed to evidence and perspectives generated outside the traditional clinical settings, which are essential to advance implementation science.

**Methods:**

Semi-structured qualitative interviews were conducted with 20 individuals, selected through purposive snowball sampling. Respondents involved representatives of three public and four private funding agencies in the Netherlands. Interviews were conducted in-person or virtually, audio-recorded and transcribed verbatim. Data extraction and data analysis were conducted using an iterative abductive thematic coding approach on the basis of the methodology of Timmermans and Tavory.

**Results:**

The strategies and related challenges of public and private funders of dementia research were clustered into three themes: “dissemination”, “implementation support” and “research ecosystem capacity-building”. Strategies for dissemination and implementation support were facilitated through brokering knowledge and providing financial incentives, procedural guidance and action mandates. Public and private funders contributed significantly to research ecosystem capacity-building through strategies such as establishing research consortium models, implementation training programs and professional connective networks. Results suggested that both types of funders are guided by distinct value systems and contribute different resources and expertise to the D&I process. Collaborative capacity between public and private funders was hindered by D&I role ambiguity and conflicting value systems, which emphasizes the lack of insights in how and when to engage each type of funder in D&I.

**Conclusions:**

This study provides contextual insight into the opportunities to invest in developing D&I professional competencies and leveraging strategic public–private partnerships to optimize D&I processes. Future research could develop this research ecosystem concept to overcome persistent contextual D&I challenges.

**Supplementary Information:**

The online version contains supplementary material available at 10.1186/s12961-025-01440-7.

## Background

Globally, the implications of an ageing population and increasing rates of dementia create demand for adaptable and scalable care solutions for people with dementia and their caregivers [1]. By 2040, the Netherlands is projected to experience a significantly increased social and economic burden, with the population of people with dementia reaching 520,000 and national dementia care costs rising to 15.6 billion euros [2]. The Netherlands participates strongly in international initiatives, such as the EU Joint Programme–Neurogenerative Disease Research, contributing to strengthening international dementia research [3]. The Dutch government previously allocated €65 million to fund research in the *Memorabel* programme (2013–2020) in response to the projected demand for high-quality dementia care [4]. The final programme evaluation emphasized challenges with research project fragmentation and research uptake due to funding discontinuation and limited stakeholder engagement with practice and industry [4]. This led to the establishment of the Dementia Research Programme (DRP) (2021–2030), which allocates €140 million through consortia models to strengthen fundamental research, risk reduction, diagnostic tools, innovative therapies, early onset dementia and knowledge transfer [5]. Consortia models connect traditionally siloed academic research projects to industry and societal partners via public–private partnerships [6, 7]. Consortia models also enhance efficient research practices and emphasize the knowledge transfer from research to practice and policy, also referred to as research dissemination and implementation (D&I).

Implementation science maturity in the field of dementia research is currently low and requires contextual evidence to inform research uptake [8, 9]. The complexity of dementia research implementation is also enhanced by an integrated multidimensional care approach for people with dementia, which covers the social, care, and welfare domains [10, 11]. Implementation science provides theoretical and conceptual guidance to reduce research D&I complexity and strengthen evidence-based decision-making capacity in research systems [12, 13]. However, implementation science research has predominantly focussed on implementation determinants and strategies at the individual and organization levels, which can entrench a linear reductionist view of implementation determinants and overlook the broader external factors that shape clinical implementation outcomes [13]. Implementation science research, particularly conducted at the systems level, is needed to increase understanding on how to develop more efficient research D&I practices and improve research uptake [14].

The systems level research D&I process is partially facilitated through integrated knowledge translation (IKT) activities, such as the active involvement of “knowledge end-users”(e.g. clinicians, research funders) in creating relevant and useful research [15]. IKT principles suggest that strategic engagement of end-users at each phase of the D&I process can systematically address the contextual D&I challenges [16, 17]. The research D&I process can be abstracted into multiple iterative phases, including (1) research co-creation, (2) research dissemination, (3) contextual assessments and selection of appropriate D&I strategies, (4) research (clinical) implementation and (5) monitoring and evaluating research D&I outcomes to drive scale-up and continuous improvement efforts [18]. Given the demand for systems-level evidence, perspectives from systems level actors are needed, such as those of the research funder.

The role and contributions of the research funder in research implementation has been explored in several studies [19–21]. For example, Van der Linden [22] surveyed 31 public and private research funding agencies across 12 countries to characterize and categorize their D&I activities. Those results determined six main practice areas: release of findings, dissemination, knowledge exchange and partnering, building capacity and infrastructure, implementation and implementation research [22]. Further, Leeman et al. [23] broadly classified funders’ research D&I strategies into “dissemination”, “capacity-building” and “scale-up” clusters. However, there remain significant gaps in evidence. First, the precise strategies and related challenges of research funders at each phase of research D&I process are unclear. Next, the activities of public and private funders, used to enhance co-funding collaboration for research D&I, are ambiguous. The ambiguity complicates the organization of D&I roles between public and private funders collaborating in co-funding arrangements within research consortia models [7]. Additional empirical research on the specific strategies and challenges from the research funder perspective is needed to generate contextual evidence that detangles D&I process complexity [5, 24].

This study explores the research D&I process from the perspectives of public and private dementia research funders in the Netherlands. This study’s overarching aim is to contribute contextual evidence (i.e. research D&I strategies and challenges) that clarifies the roles and boundaries between public and private dementia research funders and guides them in planning and executing research D&I. This approach translates the value of implementation science knowledge and tools beyond clinical settings, thereby improving research uptake through identifying and addressing organizational and systems determinants [25]. This is in line with the plea of Chambers and Emmons [26], who determined that further evidence and perspectives, generated from beyond traditional clinical settings, is needed to advance implementation science maturity and accelerate the use of research in practice and policy. With conceptual guidance from the six D&I practice areas [22], this study explores two central questions:

1. What main activities and strategies were performed by public and private funders of dementia care research to facilitate research dissemination and implementation?
2. What related organizational and external challenges did public and private funders encounter in facilitating research dissemination and implementation?

## Methods

### Setting

**Fig. 1:**
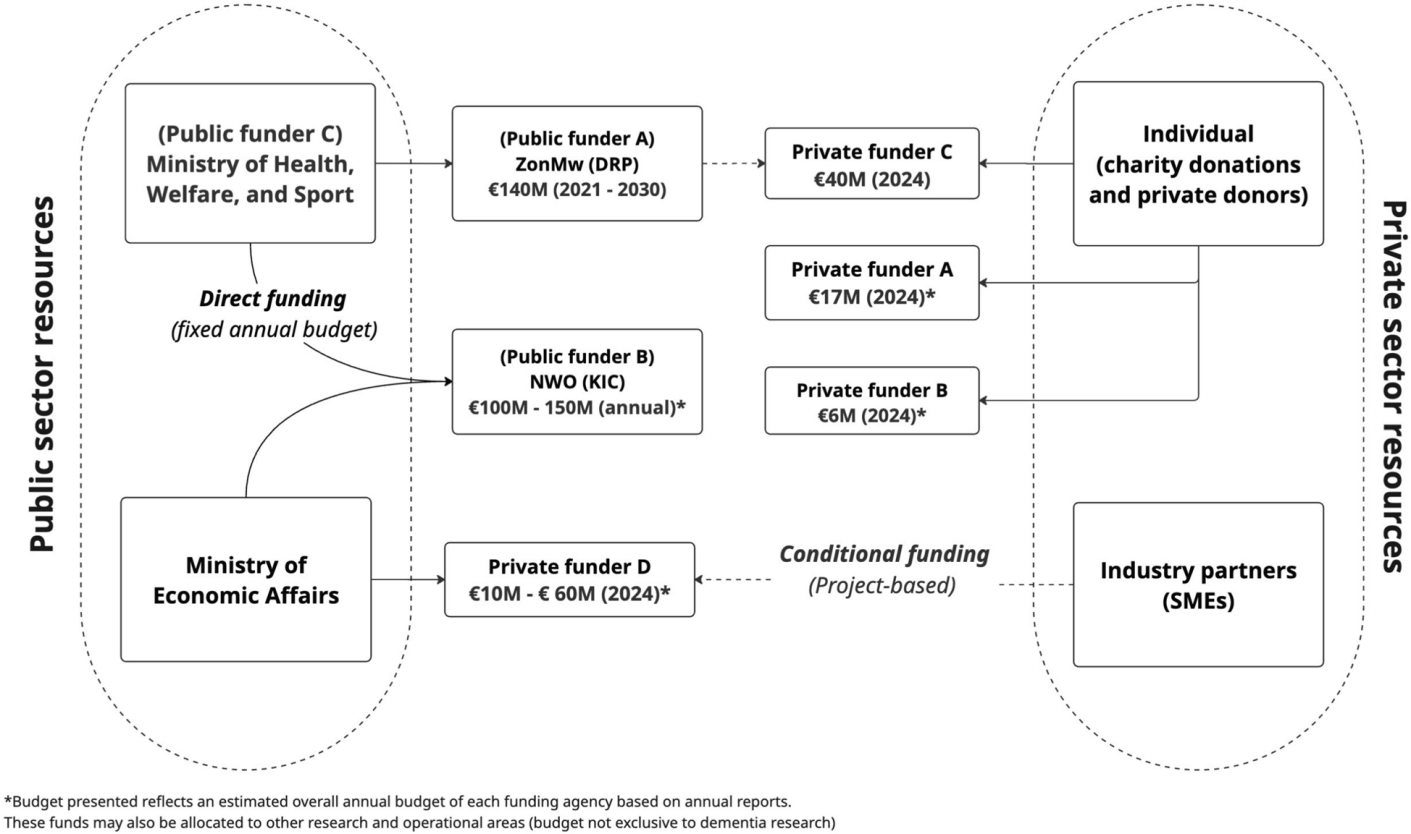
Funding streams of dementia research funders in the Netherlands

Dementia research in the Netherlands is conducted primarily by academic researchers based in public universities and research centres [27]. Research funding is allocated to these centres through public and private funders [2]. In the Netherlands, the National Dementia Strategy (2021–2030) guides dementia research agenda priorities by outlining investment areas and thematic focusses, primarily in research innovation and implementation [2]. The Ministry of Economic Affairs and Ministry of Health, Welfare and Sport receive direct funding from the central Dutch government and are tasked with allocating funding to public and private research funders. The Dutch Organization for Scientific Research (NWO) and The Netherlands Organization for Health Research and Development (ZonMw) are public funders, tasked with allocating €140 million through the Dementia Research Programme (DRP) 2021–2030. This focusses on strengthening areas of basic research, diagnostics, risk reduction, technological innovations and young onset dementia, as well as integrating research, care and education [5]. Public and private research funders also construct co-funding partnerships to foster collaboration and share responsibility, risk and ownership [7]. Cross-sector involvement with private (industry) partners and private research funders is also encouraged, notably through participatory research infrastructure [28] and public–private partnerships in research consortia [29]. Consortia models also engage nonprofit organizations, such as Alzheimer Nederland, in co-funding and public engagement. This organization of stakeholder groups also allows each group to contribute on the basis of their resources and areas of expertise, strengthening research D&I outcomes at each phase of the process. Public and private funders obtain financial resources from a variety of sources, as depicted in Fig. 1.

### Participant recruitment

Participants were purposively selected on the basis of a strict criterion to ensure external validity. Participants were included if employed by either a public or private dementia research funding agency in the Netherlands. Additionally, participants must primarily oversee research funding, dissemination and/or implementation, serving as program officers, managers or similar roles. Participants were excluded if they were not employed by a dementia research funding agency or directly involved in structuring and operationalizing research funds (such as administrative staff).

The study research team, comprising a PhD candidate and three university professors, identified potential funding agencies to include in this study through their professional networks and through referrals from prior working relationships with Dutch dementia researchers [27]. Seven funding agencies (three public and four private) were identified using each organization’s website, and respondents were contacted through email, phone or LinkedIn with individualized introductory messages. All contacted staff agreed to participate. Through snowball sampling, participants introduced other relevant colleagues and provided introductory emails on our behalf. Referrals were restricted to individuals who met the predefined inclusion criteria. To reduce the potential for sampling bias, participants were drawn from both public and private research funding agencies and represented a variety of roles across different organizational levels (project officers, managers, directors). Participation was voluntary and confidential to reduce professional pressure and bias. Participants brought varied professional backgrounds – including social, medical and implementation expertise – ensuring a representative sample of the activities and challenges faced by public and private dementia research funders in the Netherlands.

### Data collection

The interview topic guide was developed on the basis of the six funder D&I practice areas (release of findings, dissemination, knowledge exchange and partnering, capacity building and infrastructure, implementation, implementation research) developed from a survey study of 31 international funding agencies [22]. These areas were selected to guide data collection given the comprehensiveness and pragmatic relevance. Specific open-ended questions were designed to explore Dutch dementia funders’ strategies that facilitate the practice areas and identify the related emerging contextual challenges (see Additional File 1: Table S1). The interview topic guide was refined with support from the original study’s first author to ensure accurate understanding and application of the practice areas by the research team [30]. The interview guide was pilot-tested in an initial interview with a pair of respondents but did not require any changes.

The first (EMZ) and last author (RH) conducted the interviews together, in-person and online via Microsoft (MS) Teams. In total, 15 interviews (5 with pairs, 10 with individuals) were conducted with staff members in Dutch dementia research funding agencies between May and July 2024, each lasting 60–75 min. Each interview was audio- and video-recorded (via MS Teams), transcribed and pseudo-anonymized. Respondents were provided a copy of their own interview transcript to provide clarification or retract statements. None of the participants withdrew from the study. Interviews were conducted until data saturation was reached (i.e. responses were repeated).

### Data analysis

Each interview was transcribed verbatim and reviewed by the first author (EMZ) to generate in-depth familiarity with the content and initial insights on the potential patterns in the transcripts. Data extraction was performed by the first author manually using Microsoft (MS) Word to create a coding framework to organize data. Data extraction and data analysis were conducted using an iterative abductive thematic coding approach on the basis of the methodology of Timmermans and Tavory [31, 32]. Accordingly, data were collected iteratively until interpretive patterns were well developed and did not produce any new divergent insights. This abductive approach iteratively executed inductive and deductive data extraction and analysis across three stages.

In the first stage, the first-order (open) codes were labelled throughout each transcript to ensure the intended meaning was accurately captured and conveyed [33]. This allowed the authors to inductively identify emerging concepts or ideas that may transcend the conceptual boundaries of any singular practice area [34]. These open codes captured the specific activities undertaken by research funders and the related challenges they encountered throughout the research D&I process.

In the next stage, second-order (axial) codes were abductively developed and discussed in the whole research team on the basis of the six practice areas [22] and the commonalities identified across the inductive open codes. Initially, the six practice areas were used to organize the inductive open codes (i.e. activities and related challenges) and create deductive axial codes (i.e. overarching strategies) for further analysis. However, in their team discussions, the authors recognized that several open codes (i.e. activities) fit in multiple practice areas. This prompted the authors to reconstruct the grouping of open codes, creating inductive axial codes (i.e. strategies) that appropriately capture the linkages and commonalities between open codes (i.e. activities).

Lastly, selective codes were generated by grouping axial codes (strategies) into thematic relational clusters (strategy clusters) to produce higher-level constructs used for implementation theory-building [33, 35]. All authors actively reflected and discussed these axial and selective codes to ensure reliability and internal validity [36]. All authors were involved in developing and refining the themes reflected in the final manuscript. The coding framework can be found in Additional file 1: Table S2. The qualitative reporting in this study was guided by the COREQ checklist in Additional file 1: Table S3 [37].

### Ethical considerations

This study was approved by the Research Ethics Review Committee at Erasmus University Rotterdam (ETH2324-0620). Participants signed informed consent forms outlining the study’s scope and intended data use, ensuring transparency and protecting their privacy rights.

## Results

A total of 20 respondents, recruited from three public and four private research funding agencies, participated in the interviews. Individual respondents included 10 entry-level program officers (1–5 years of experience), 7 senior-level team managers (5–10 years of experience) and 3 director-level agency leaders (more than 10 years of experience). The primary functions of participants spanned research grant and project management (12), public dissemination and advocacy (4) and strategic coordination (4). Of these 20 respondents, 11 were recruited from a public funding agency and 9 were from a private funding agency.

The strategies and related challenges of public and private funders were clustered into three themes: “dissemination”, “implementation support” and “research ecosystem capacity-building”. The dissemination and implementation support clusters included the original activities in the release of findings, dissemination and implementation practice areas. Strategies found in these clusters were selected and carried out by funders on a project basis. In addition, dissemination and implementation support strategies were further delineated into “direct” and “indirect” strategies on the basis of the activities of each strategy. Indirect strategies enable funders to achieve the intended outcome by guiding, incentivizing or mandating action from an intermediary body (e.g. research teams). Direct strategies enable funders to achieve their intended outcome (e.g. dissemination) without involving any intermediaries. This distinction provided further conceptual clarity to guide the strategic selection of strategies that facilitate research D&I process planning and execution. Additionally, research ecosystem capacity-building strategies encompassed research funders’ direct activities in the “knowledge exchange and partnering”, “building capacity and infrastructure” and “implementation research” practice areas. These strategies also contributed to clarifying systems level structures and processes, which also strengthen the outcomes of project-based strategies.

## Strategies for dissemination and implementation of dementia research funders

### Dissemination strategies of research funders

The dissemination cluster encompassed strategies and activities associated with release of findings and dissemination practice areas. Release of findings activities assumed that research findings will diffuse to relevant audiences and be accessed by end-users autonomously, whereas dissemination activities assumed that the research results should be “translated” and “tailored” by funders to fit the interests and contextual needs of their targeted audiences.

Public and private funders employed indirect dissemination strategies, stratified into three typologies: “incentive-based” (i.e. additional funding), “mandate-based” (i.e. requirements) and “guidance-based” (i.e. frameworks, tools). Public funders incentivized researchers to disseminate research by providing research subsidies, which also “mandated” Open Access publications and data sharing under Findable, Accessible, Interoperable and Reusable (FAIR) principles. They provided guidance (e.g. Dutch Research Council NWO Impact Framework) to help research teams plan for dissemination and engage external research stakeholders and end-users. Private funders also incentivized research dissemination by providing PhD thesis awards to researchers and requiring research end-users’ involvement in the initial project planning stages.

*But one thing that is kind of hard for us is that our subsidy isn’t allowed to be used for broader dissemination. We can only finance activities that are scientific dissemination, scientific papers, scientific congresses, academic visits, etc. (…) But we also try to push more and more to include the end user in their research from the start* (respondent 19; private funder).

Public and private funders also employed direct dissemination strategies, primarily involving knowledge translation and public–private engagement to widen research accessibility to targeted audiences. The range of knowledge translation activities included (1) translating the evidence from academic jargon to layman language, (2) synthesizing research findings into evidence briefs and (3) adapting evidence from research and creating multimodal formats (e.g. videos, booklets) to scale dissemination and reach. Public funders synthesized research findings into decision-making tools (i.e. evidence synthesis) for policymakers and created multimodal media to share findings with patient groups. Private funders translated research findings for industry sponsors, individual donors, patient groups, practitioners and policymakers (i.e. advocacy teams). Both public and private funders facilitated cross-sector matchmaking events (e.g. Mix and Match, Alzheimer Cafes) to share translated evidence and create opportunities that foster cross-domain collaborations along research ecosystem stakeholders. Private funders furthered cross-sector engagement by engaging policy advocates and leveraging professional networks from external partners to enhance research dissemination. Table 1 provides further clarity to delineate between the dissemination activities of public and private funders.

**Table 1:** Dissemination strategies of public and private research funders

Nature of funders	Main strategies identified from both public and private	Sector specific details
Public funders	(1) Indirect dissemination strategies: • Incentive-based (i.e. awards) • Mandate-based (i.e. requirements) • Guidance-based (i.e. frameworks, tools) (2) Direct dissemination strategies: • Knowledge translation • Public–private (research ecosystem) engagement	• (Indirect) Requires Open Access publications resulting from funded research (FAIR principles) • (Direct) Translate evidence to decision-making tools to support policymaking • (Direct) Translate evidence to multimodal formats for public end users
Private funders		• (Direct) Engage champions (i.e. ambassadors) to share research to policy context • (Direct) Translate evidence for individual donors, patient groups, policymakers • (Direct) Share research through (professional) networks of external partners with relevant audiences (e.g. healthcare institute, Memory Clinics)

*We were searching for an [ambassador], and we asked her to connect organizations and to make things change more quickly about case management, dementia, about daycare activities, and about housing. So she talked to a lot of people. And she organized a lot of conferences and made a report after a year (…) I think it helps that a woman of that stature did what she did and talked to people. She was very good in convincing and connecting organizations and opposites [sides]* (respondent 14; private funder).

*We tried to reach the general public more on social media. I think there are other partners that can reach other target audiences better, such as healthcare professionals. For example, Zorg voor Beter (Care for Better), the Netwerk Kennissteden Nederland (Network Knowledge Cities Netherlands) and the Dutch Memory Clinics. We can use those partners’ [networks] to reach the health care professionals* (respondent 12; private funder).

*We try to collaborate with researchers to inform Members of Parliament and politicians. Not very often, but we do. And of course we invite them to the media to tell their stories about how they do things and about the results. We have a TV show with researchers telling about what they are doing and why it’s important for people with dementia (…) we like those researchers who can talk about what they are doing and their results in a way “normal” people can understand* (respondent 14; private funder).

### Implementation support strategies of research funders

Funders’ implementation support strategies encompass the implementation practice area. Results suggest that funders are not involved with the actual implementation of research findings into implementation settings (e.g. nursing homes, hospitals). More accurately, funders facilitated implementation support through indirect and direct strategies.

Indirect implementation support strategies of funders were also incentive-based (i.e. additional funding), mandate-based (i.e. requirements) and guidance-based (i.e. impact frameworks). Public funders offered incentives through implementation subsidies, such as Dissemination and Implementation Impulse (VIMP) grants and impactful program awards (ZonMw PEARLs). Knowledge vouchers were also issued for research teams to hire implementation specialists that build implementation capacity. Public and private funders required research implementation and impact plans, guided by Promoting Responsible Research Practices (BVO) criteria, to promote equitable societal benefit from research. Public funders required structured implementation plans at the grant application stage and provided impact modelling tools (e.g. NWO Impact Focus Approach [38]) as guidance. In contrast, private funders used a proactive and adaptive approach to guiding, refining and tailoring research impact and implementation plans on the basis of implementation progress, such as a digital health product’s technology readiness level. This enabled private funders to anticipate potential challenges to implementation (e.g. procedural barriers), respond with timely and appropriate implementation support and efficiently steer research implementation.

*We had a big part in dissemination of the results, putting on some nice texts on our websites or LinkedIn, funding certain parts, but now we try to be more proactive (…) we want to say to them, “Hey, this looks interesting. Did you already think about certain implementation stages?” So we try and steer more actively* (respondent 17; private funder).

*Researchers have to complete an application form, which is specifically built according to that impact plan approach. So every researcher has to complete that application form, fills in parts of that impact plan approach, basically. With, of course, the advice on our side, to really start with the end in mind. And then, reason back* (respondent 8; public funder).

The direct implementation support strategies, employed by public and private funders, required their active contribution to facilitate project-based research implementation. Strategies primarily included (1) brokering partnerships other research D&I stakeholders to identify implementation and scaling opportunities (e.g. across municipalities and across health and social domains) and (2) leveraging innovation systems expertise and valorization pathways for dementia research. Public funders primarily fostered professional relationships across the research ecosystem to connect health and social domain research partners with evidence, such as embedding and scaling dementia research findings within municipal health services for broader impact. They also performed activities that focussed on four “productive interactions” that guided implementation support, including stimulating research co-ownership between public and private funders, connecting with research ecosystem stakeholders, making usable products for societal benefit and developing effective dissemination and implementation strategies.

*In every program and project, you will see metrics on either productive interaction, in these four categories, or an open question: Who is going to profit by these results? How are you going to reach them? Is there a role for [funder]? Can we help you? Do you need extra money for an extra grant for implementation?* (respondent 6; public funder).

Next, public funders were legally restricted from supporting projects that commercialize, or profit from, their research investments. In contrast, private funders applied innovation management principles to promote valorization and innovation in research funded by their investments. They leveraged valorization structures, including incubators, accelerators (e.g. Health Impact Accelerator) and technology transfer offices, to advance dementia research accessibility and commercialization. Private funders also engaged private sector stakeholders (i.e. investors) to connect academic research with industry, securing sustainable financing, enhancing business models, and creating pathways for scaling dementia research. Table 2 provides further clarity to delineate the implementation support activities of public and private funders.

**Table 2:** Implementation support strategies of public and private research funders

Nature of funders	Main strategies identified from both public and private	Sector-specific details
Public funders	(1) Indirect dissemination strategies: • Incentive-based (i.e. awards) • Mandate-based (i.e. Promoting Responsible Research Practices criteria) • Guidance-based (i.e. frameworks, tools) (2) Direct dissemination strategies: • Knowledge translation • Cross-sector (research ecosystem) engagement	• (Indirect) Dissemination and Implementation Impulse grants; Impactful Program Awards; Knowledge vouchers (for implementation specialists) • (Indirect) Structured implementation plans and modelling tools • (Direct) Establish professional relationships across systems (education, health, welfare) to broker connections • (Direct) Four productive interactions (cross-sector co-funding; connecting with research ecosystem stakeholders; making usable products; and developing effective D&I strategies)
Private funders		• Proactive, adaptive approach to implementation planning, based on research progress (e.g., product Technology Readiness Level) • Valorization and commercialization of research through incubators, accelerators, technology transfer offices • Seeking private investors to provide sustainable financing opportunities

*There is a lot of discussion. In principle, everything that is created by [public] money should be openly available, should be reported on, it should be put on our website and included in all our products and databases. (…) We want implementation, but we are not allowed to commercialize it. Sometimes, it makes you think that if we didn’t need to do it [implementation], we wouldn’t exist. This is also a big discussion on this state support, which makes it that you [public funders] are not allowed to support commercial organizations from Europe, as a public funder. But on the other hand, if it would be done by the market, we wouldn’t need to exist* (respondent 6; public funder).

*A lot of colleagues of mine now have knowledge on valorization. Valorization goes over the whole aspect of research. It’s all about knowledge utilization. And of course, I have some knowledge of patent filing, but I don’t have the knowledge of patent filing that a Knowledge Transfer Office has at an academic centre. What we tried to do is support and facilitate researchers the best we can. So we try to connect them with other parties that actually can help them further on the road* (respondent 17; private funder).

*So we’re going to form a panel of external investors and they are also going to rate the project on their market viability (…) there’s also the idea or the goal to start a phase three so that projects that come from the phase two projects can also apply for future follow up funding. So we really try to guide the projects and the innovation from the start from different levels, phase one or phase two, and then can go on to the to the next funding possibility* (respondent 19; private funder).

### Research ecosystem capacity-building strategies of research funders

Research ecosystem capacity-building strategies include activities under the knowledge exchange and partnering, building capacity and infrastructure and implementation research practice areas. Funders of dementia research employ these strategies at the system level to strengthen infrastructure and processes that support the entire dementia research D&I process, simultaneously enhancing the project-based dissemination and implementation support strategies. Funders reported capacity-building strategies as direct strategies that improve research implementation outcomes by iteratively and continuously strengthening the formal research ecosystem infrastructure and resources. Such strategies focussed on advancing the infrastructure of human capital (via training), professional (collaborative) networks and research governance structures, which all contribute to improved research D&I outcomes.

First, funders strengthened the capacity of human capital by broadening their internal (staff) professional D&I competencies. Cross-professional training, such as for advocacy and lobbying, research communication and brokering and cross-sector partnership management, was provided to public and private funders. These skills allowed funders to improve their ability to act as knowledge brokers and boundary spanner between academics and industry stakeholders. Public funders also strengthened (external) human capital by building an implementation science training program (Implementation Science Practitioner Fellowship) that aims to formally train external researchers and practitioners and expand the knowledge and scale of D&I professionals in the Netherlands. This develops the depth and scale of D&I expertise available externally to support research practices.

*We noticed that it is not enough to ask certain questions on implementation. We also need to build the infrastructure… help to build the infrastructure of people and networks that is needed to do that implementation. (…) We are becoming a little bit stricter when it comes to implementation. But we also noticed that we need to help to fill that knowledge* (respondent 7; public funder).

Next, funders strengthened the capacity of professional networks by creating research D&I implementation pathways and collaboratives to systematically design and mobilize evidence across the research ecosystem. Public funders, researchers and practice professionals established a formal network of implementation science professionals (Netherlands Implementation Collective) to enhance connectivity within the implementation science community and to build connectivity across the wider professional D&I support infrastructure. Public and private funders strengthened “professional networks” by creating formal cross-sector consortium partnerships (e.g. ABOARD consortium) to stimulate research use beyond academia. These networks were established by funders to help align research ecosystem stakeholder interests, determine research agendas and set research funding programs.

*There are few people within the university, in the technology transfer centres, that are specialized in implementation. That’s of course also why we put it on the national agenda to have more capacity in this field. We started with founding the Dutch implementation collaboration. So it’s broader than only valorization centres. The specific corner of our organization focusses on fundamental research and E-health. Those domains of research talk more about valorization instead of implementation* (respondent 5; public funder).

Lastly, dementia funders strengthened research D&I capacity by building research governance structures that improve the organization and governance of research projects and their outputs. Such structures include the public–private research consortia model (e.g. DEMPACT consortium), which help reduce research fragmentation and promote knowledge connectivity across sectors, shared risk and ownership over research and expertise exchange between academia and industry. Funders also developed “monitoring and evaluating structures”, such as implementation evaluation metrics and theory of change models, to audit and assess research D&I outcomes. This strengthens their own ability to organize D&I efforts, monitor implementation outcomes and adapt to external demand for implementation support. Table 3 provides further clarity to delineate the research ecosystem capacity-building activities of public and private funders.

**Table 3:** Research ecosystem capacity-building strategies of public and private research funders

Nature of funders	Main strategies identified from both public and private	Sector-specific details
Public funders	(1) Strengthened the capacity of “human capital” • Internal (funder staff competencies) • External (workforce competencies) (2) Develop professional networks • Implementation support practitioner collaboratives • Public–private consortium network (3) Research governance structures • Public–private research consortium models (shared ownership, risk, responsibilities) • Monitoring and evaluating structures	• Establishing formal training programs for implementation support practitioners • Expanding the core competencies of funders to include project D&I planning and management, cross-sector collaboration, and knowledge brokering • Construct professional (expert) networks (e.g. Netherlands Implementation Collective) • Developing monitoring and evaluating criteria for implementation projects
Private funders		• Use infrastructure (e.g. volunteer network) to monitor and evaluate regional dementia conditions • Expanding the core competencies of funders to include research brokering, research advocacy and partnership management • Joining public–private partnerships to influence research agenda development

*We have also trained our colleagues to facilitate big matchmaking and collaboration sessions. We are very much interested in how we can help [funder] give the information, but also help with getting partners to collaborate with each other* (respondent 9; public funder).

*We had contact with [other funders] to create the Call text, to make arrangements about who funds what. Since they want to incorporate companies, but the government cannot fund free market companies, they have to be efficient on that. So they cannot fund all the costs of companies. It’s a percentage of 50–60%, and our money can match this funding so we can pay for the company’s bill because we are a NGO [not restricted by government budget]* (respondent 16; private funder).

## Challenges of dementia research funders for dissemination and implementation

Public and private funders reported several distinct challenges relating to each thematic cluster. For the dissemination cluster, funders observed that research teams were underutilizing the funders’ available dissemination channels (e.g. newsletters, blog posts, media), which led to low engagement with the public. Additionally, funders identified challenges in reaching broader audiences through their current channels, particularly vulnerable groups such as older individuals with low digital literacy and limited access to technology.

*For communication, the problem is that you cannot always reach the people that you want to reach, but you reach the people that want to read it. I think that’s a really big issue. How do you reach the people that really need to know it? And I think that’s in general challenging. But there are just hard to reach target audience and especially for older people…maybe not that used to the Internet. How do you reach them?* (respondent 12; private funder).

*I want to develop new strategies to connect researchers and implementers. Because people doing research only think about articles and presentations. But this is not enough… because people working in dementia care, they don’t go to those presentations. They don’t read those articles. I think those consortia researchers even have to change their ways of doing research. Participation of people with dementia, carers, health carers, welfare carers… they have to participate along. So we have to think of other ways of doing research* (respondent 14; private funder).

In the implementation support cluster, public and private funders faced two major challenges in providing project-based support. First, the unclear research D&I roles amongst public funders, private funders and research teams led to conflicting expectations regarding each party’s respective responsibilities and competencies. For instance, the absence of implementation support guidelines, which clarify such responsibilities across the research D&I process, has led to a reactive approach to implementation support. Without clear determination of stakeholder roles across the research D&I process, funders struggled to select appropriate implementations support strategies and deliver effective implementation support. Next, there is low engagement from researchers with interdisciplinary expertise and resources to support research D&I. Funders provided implementation support infrastructure, including innovation valorization structures and implementation support vouchers, to enhance D&I efficiency and reduce resource waste within research teams. However, the limited engagement from researchers reduced the reach of evidence to diverse audiences and impeded public access to the benefits of research.

*We try to stimulate the researchers. We are funding research. So, we try to stimulate the implementation. (…) But the field will have to take it on. Also, if you want it to work for the coming years, there has to be some money [invested] from the organization itself, because otherwise, after one year of money from [funder], then who is going to pay for it then? They have to look at how they are going to pay for this. We can do things to stimulate it or to give the first push, but the field has to take it…* (respondent 1; public funder).

*There’s a pathway from the bench to bedside. And I think you cannot expect that the researchers at the bench bring it to the bed. It’s a chain and we think it's important that you talk to the right person, and that they think forward, to whom I can hand it over. Most of the time, what happens is [that] they did something at the bench. They discovered it. And then they started over again with another project* (respondent 16; private funder).

In the research ecosystem capacity-building cluster, public and private funders encounter complexity-related challenges resulting from (1) the low maturity of current research D&I infrastructure and (2) conflicting value systems in public–private partnerships. First, complexity-related challenges stemmed from the low maturity of research D&I infrastructure, such as limited expertise (workforce capacity), inadequate structural financing for implementation and insufficient research impact evaluation mechanisms. These limitations reduced funders’ ability to systematically select and employ strategies that enhance D&I outcomes.

*It takes a lot of attention or time to start this program in a good way, and then we have too little time or attention for really following up [on] the results and making sure that it reaches its impact. So that’s one of the challenges. And the other thing is, that we do a lot of projects. So it’s very difficult to see all these connections between this problem, and then there are a lot of connections* (respondent 5; public funder).

Further, these limitations hinder the ecosystem’s capacity to monitor, evaluate, and improve the outcomes of current D&I strategies, contributing to further implementation uncertainty and complexity.

*One of the questions that we were asked, was how the program contributes to the national dementia strategy. Specifically on the [goal that] 80% [of people with dementia] has access to meaningful activities etc. We said that we can’t do that. There is no direct link between what we’re trying to achieve in the field and the access [to research impact] that people have. (…) We can try to colour them with stories from the project [results] and the municipalities with their experience within the program* (respondent 4; public funder).

*It is a challenge because we want to be able to say “this is the impact of our project”. But the things that come out of projects that are measurable are the amount of patents and amount of publications. So we have a whole list where they need to fill in. “What are the outputs of your research?” But it is quite hard to measure the societal impact and also the economic impact. So they need to address this in their application. But there’s not yet a real strong way for us to assess all the impacts from our project and it is something that we try to develop and make it better. But we have some numbers, for example, on publications. But we all know that it's not the best indicator* (respondent 19; private funder).

Next, in the dementia research field, public–private partnership consortia models are research governance structure that connects cross-sector interests to advance dementia research impact. However, funders experience challenges within these partnerships, primarily relating to (1) strategic stakeholder engagement and (2) value systems (mis)alignment for goal and agenda setting. Strategic stakeholder engagement in public–private partnerships is vital for research ecosystems to thrive, but this comes with unique challenges. For example, public funders face legal and regulatory barriers, such as EU State Aid rules that restrict the commercialization of publicly funded research. Public–private co-funding partnerships enable publicly funded research products to leverage commercialization strategies supported by private funders.

However, these partnership models are new in the Dutch dementia research context, and funders are often challenged by the conflicting value systems between stakeholders. Funders characterized value systems in this context as the set of values, priorities and objectives that motivate individuals and institutions from different sectors and settings to be involved in research D&I. For instance, funders reported that practice setting stakeholders often prioritized simple, feasible, effective research products, whereas academic stakeholders, primarily responsible for producing research, may be evaluated on the basis of innovation and scientific rigour. This creates tension amongst stakeholders and poses challenges for funders to set research agendas that align practice and academic value systems.

*But this is the key of what is happening also because sophisticated research and results and interventions are more quickly highly rated… and people who develop more simple interventions which people with dementia like, they’re not for* The Lancet *or for high rated journals. This is a problem* (respondent 14; private funder).

*I know that one of the problems, for example, for medical products is that it’s under European legislation and you have to have a certain certification, which also accounts for very small interventions like an app. And this whole trajectory also involves legal people from Brussel, which counts €800 per hour for that advice. And the talk about the cost of such territory is about €150 000 or something. So then they’re not able to get this accreditation. So they’re not allowed to go to the market. So that’s one of the things that I know that are really blocking the implementation or the use of this knowledge* (respondent 5; public funder).

*If a party will generate money from it, then we have a problem with the public money that comes from the Ministry of Health. (…) We have these strict regulations about that you cannot earn money with it and have an advantage over other organizations in the field. (…) we give money to one organization, and they will have an advance on the market and the other organization not… Then, it is stated that it is prohibited* (respondent 1; public funder).

## Discussion

The strategies and challenges identified in this study provide contextual nuance and depth that informs research D&I processes at the system level. Findings from this study produced valuable theoretical and practical contributions to advancing D&I research, informed through evidence from the dementia research domain. The main D&I strategy clusters identified in this study include dissemination, implementation support and research ecosystem capacity-building. These findings closely align with the dissemination, capacity-building and scale-up strategies of support system actors, as proposed by Leeman et al. [23]. Each of these strategies require contributions from diverse stakeholders. This aligns with the principles of integrated knowledge translation (IKT), which encourages the involvement of research funders, research teams, practitioners and policy actors in the research co-production and D&I process [39]. Additionally, this study also revealed that there are clear interdependencies between institutions (e.g. research funders, research teams) and across sectors (e.g. public and private funders) at every phase of the research D&I process.

Given these findings and insights, this study proposes a research ecosystem approach to frame and structure the IKT activities of research D&I stakeholders. This conceptualization builds upon Adner’s innovation ecosystem concept, commonly utilized in the fields of entrepreneurship management and innovation science [40]. The innovation ecosystem concept contains structures and processes, unified through an ecosystem-as-a-structure approach, that frame and align the activities, actors, positions and links involved in value creation [41]. There are several clear commonalities between the innovation ecosystem and this study’s proposed research ecosystem (see Table 4 for more details). Accordingly, the research ecosystem concept could also be operationalized using the ecosystem-as-a-structure approach and used to guide funders in explicating interdependencies and organizing boundaries between the peripheral stakeholder groups involved in value creation [41]. Tools and models, such as the Ecosystem Pie Model [42], are available to structure innovation ecosystems and support planning. This model includes several elements that contribute to understanding and capturing “value” in the ecosystem, such as the actors and resources involved, activities performed, value addition and value creation [42]. These tools may be adapted for use in the dementia research ecosystem context to specify, frame and coordinate actors’ roles, responsibilities and resources across the phases of the D&I process.

**Table 4:** Innovation ecosystem and research ecosystem commonalities (definitions and examples)

	Traditional definition of each element applied in the *innovation ecosystem* [41]	Description of each element applied in the research ecosystem
Focal firm	The entity (individual or organization) around which the ecosystem is structured	(In this study) Research funding agency
Value creation	The promised benefit that the target of the effort is to receive (i.e. intended outcome of activities)	Dissemination outcomes Implementation outcomes Wider (health, social, educational) impact outcomes
Activities	Discrete actions to be taken for value proposition to be created	Dissemination strategies and activities Implementation strategies and activities Implementation support strategies and activities Capacity-building strategies and activities
Actors	Entities (individuals or organizations) that undertake the activities	Research teams; (other) funders; patient representative groups; policymakers; implementation settings (nursing homes, hospitals); media outlets
Positions	Positions refer to where actors stand in terms of influence, resources and responsibilities within the ecosystem	Funders occupy a central role in research agenda setting and capacity building; peripheral role in direct research implementation Researchers focus on evidence generation; peripheral role in research agenda setting and systems capacity building
Links	Links are the relationships and interdependencies between actors	Formal partnerships (consortia partnerships; public–private partnerships) Data-sharing agreements across the ecosystem

This study revealed that there was no clear consensus on the D&I roles and responsibilities of dementia research funders and research teams within the research ecosystem. This aligns with existing IKT literature that highlights the need for additional research that identifies when and how to engage each stakeholder group in research D&I [16]. Literature also suggests that a systematic approach to facilitating IKT is needed, such as by using implementation science logic models to explicate each step of the IKT process and rationale for each decision [12, 16]. Results from this study delineated the strategies of research funders into “direct” and “indirect” dissemination and implementation support strategies. This distinction provides deeper insights into the nature of research funders’ and research teams’ activities to guide IKT processes and support the development of an appropriate D&I logic model that enhances collaborative capacity [12].

**Fig. 2:**
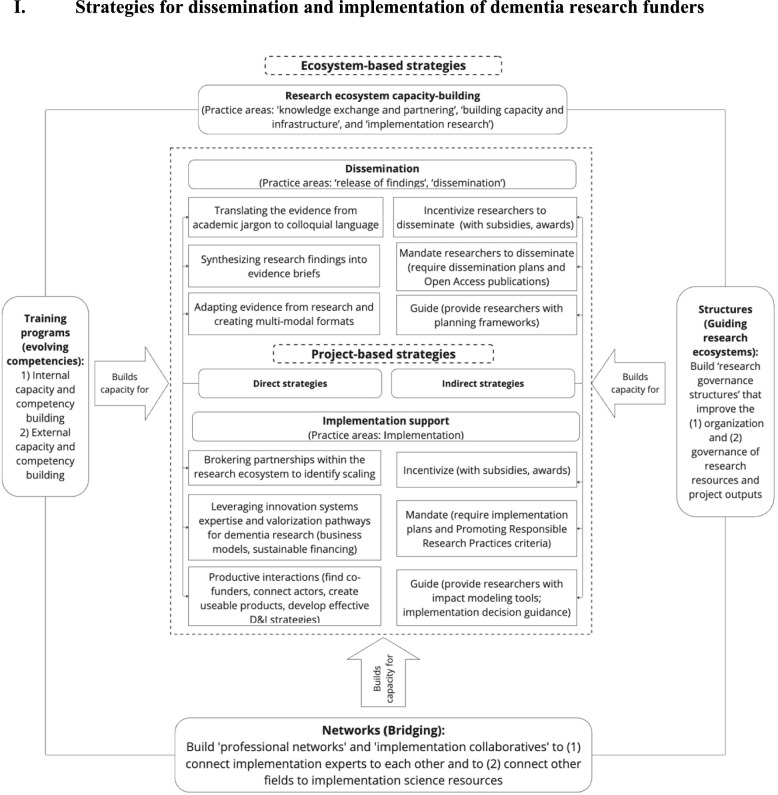
Overview of D&I strategy clusters and individual strategies employed by funders

The distinction between direct and indirect strategies provides funders with more conceptual clarity of their role in the D&I process. This aids decision-makers in D&I strategy selection and capacity-building investment agenda (see Fig. 2). Funders that employed direct strategies (e.g. evidence translation, knowledge brokering) accepted their active role in research D&I. This prompted additional investment in internal capacity building, such as by building staff competencies for managing cross-sector connections and collaborations. Noordegraaf [43, 44] argues that shifts in external demands may stimulate the “reconfiguration” of traditional professional roles and responsibilities. The national prioritization of dementia research D&I [2] stimulates research funders to “reconfigure” their traditional role and responsibilities and adopt hybrid competencies that strengthen their ability to conduct direct dissemination and implementation support activities. Such hybrid competencies include fostering intervention and implementation co-creation [45] and facilitating research ecosystem stakeholder engagement through organizing cross-boundary collaborations (e.g. public–private partnerships) [46]. Building internal hybrid competencies can strengthen research funders’ D&I capacity and position them as an implementation support hub, enabling them to play a centralized “coordinator” role within the research ecosystem.

Alternatively, funders that employed more indirect strategies (i.e. incentives, guidance) shifted D&I responsibilities towards the research teams and other research D&I ecosystem stakeholders. This outward shift of D&I responsibilities required funders to investment in the external capacity building, such as by establishing implementation support practitioners as a formal profession that complements the clinical healthcare workforce [47]. The gradual emergence of this new profession requires the timely development of context-appropriate implementation support training programs (e.g. Implementation Science Practitioner Fellowship) [48] and national D&I professional networks (e.g. Netherlands Implementation Collective) to connect and support implementation researchers and practitioners [49]. These external capacity-building activities also directly contribute to strengthening implementation science maturity, which has been hindered by a global shortage in implementation science experts and context-appropriate educational programs [50].

Further, research funding models are cyclical and are not yet equipped to meet the emerging demand for research implementation and sustainment. These may also need to evolve to meet emerging demand for research implementation funding, such as by shifting towards private funding mechanisms to bolster public research implementation [51]. Results highlighted the impact and opportunities of diverging value systems of public and private research funders collaborating within public–private partnership (PPP) consortia models. For example, public funders activities demonstrated “accountability, impartiality, transparency, and quality”, prioritizing the societal impact of research [52]. This was achieved through their “productive interactions” with society, focussed on facilitating D&I through co-creating relevant products from research with end-users [53]. Private funders more often valued “profitability, efficiency, and innovation” in research and directed resources towards research uptake and value creation through commercialization [54]. The contrasting value systems of the public and private domains may contribute to role ambiguity amongst actors in co-financing arrangements. However, there are also emerging opportunities to leverage practices to overcome respective institutional barriers.

According to public management literature, PPPs are valuable as a management structure for collaboration to reduce intersectoral knowledge fragmentation and promoting innovation and evidence uptake [55]. Successful PPPs are driven by a clear understanding of the unique strengths and competencies that each public and private actor brings to the table, coupled with a strong alignment of values and shared objectives amongst all partners [52]. For example, as identified in the results, publicly funded dementia research in the Netherlands is regulated by the European Union (EU) State Aid policies, which restrict public investment in commercial activities to protect market competition in EU Member States [56]. These policies potentially stymie innovation in publicly funded academic research and pose challenges in aligning value creation in PPPs [56]. Therefore, these unique contextual conditions require strategic organization of roles and resources of public and private actors in the research ecosystem.

Results highlighted that public–private co-funding mechanisms in dementia research consortium projects may help circumvent these State Aid limitations and offer solutions to these limited policies. The optimal role and position for public and private funders in the D&I process may be determined on the basis of the values, resources, expertise and contributions. For instance, Dutch public funders may provide subsidies for “pre-competitive research”, including fundamental research and early stage research and development [57], and offer implementation support by connecting research teams to (public) implementation settings (e.g. municipal health services). Private dementia research funders are well positioned in their professional networks to foster university–industry connections. Private funders may also provide complementary financial subsidies and offer expert guidance to transform pre-competitive research into market-ready products. Emerging research from the technology domain provides insight on this convergent approach and may help navigate valorization and commercialization of dementia research [56, 58]. However, stronger empirical evidence is needed to optimize the management of PPP resources and determine the optimal positions for public and private actors in the dementia research D&I process. Future research may consider ethical conflicts when navigating the boundaries of private research funders in public research consortia, ensuring public good remains a priority in partnership practices.

## Research implications and future direction

The study identified two key areas for further development: enhancing capacity-building infrastructure to strengthen research D&I and optimizing the roles and responsibilities of actors in the research ecosystem. First, this study determined an additional need to strengthen capacity-building, in the research ecosystem, to build dementia research D&I. This can be achieved through conducting implementation research, which did not appear in this context, potentially signifying that this is a low priority. Focussed implementation research, and “research-on-research”, is crucial for advancing the maturity of implementation capacity and enhancing societal impact. Further, capacity-building may be achieved by building large-scale connective D&I infrastructures, such as the Cancer Control Centers (ISC3) Network (USA) [59], to reduce fragmentation of roles, responsibilities and resources across the D&I process. The development of implementation science training programs, specifically for dementia research, may also strengthen D&I capacity [60]. This requires combined efforts from funders, researchers and educators to understand the D&I training needs of all implementation stakeholders to produce suitable content and to deliver training effectively and equitably [60].

Second, the research ecosystem conceptual approach is steadily emerging in IKT literature but requires empirical research and interdisciplinary collaboration to produce clear contextual insights to inform role clarity and develop decision aids and pragmatic tools (e.g. Ecosystem Pie Model). This may be achieved by using a Delphi method to create a valid dementia research ecosystem stakeholder map, build a consensus on the scope of each stakeholder’s role and contribution and identify areas of disagreement to direct future research. Further, interdisciplinary methodological research may integrate knowledge from innovation management and implementation science to build adapted tools that bolster this research ecosystem approach and reduce the evidence gap between disciplines.

### Strengths and limitations

This study may have limitations relating to the research design and data collection. First, the study used purposive (snowball) sampling to identify and select respondents. This may introduce selection (sampling) bias and reduce the generalizability of findings beyond the Dutch context. Participants were also limited to funders of dementia research, which may limit the applicability of results beyond this funding landscape. Further, the study relied on data from interviews, which may be subject to social desirability bias. Future studies may address these limitations by exploring the validity of these findings through mixed-methods approaches. Second, given the focus on research D&I through an implementation science lens, technical jargon was often used in the interviews. However, funders were accustomed to using a different set of terms to describe D&I activities. This required the interviewers to adapt their questions and adopt the funders’ terms. Data collection was conducted in English due to the language limitations of the first author. Respondents were given the opportunity to elaborate on ideas further in Dutch to mitigate miscommunication risks if this felt necessary. As all interviews were conducted by two researchers (first and last author), a native Dutch speaker was present in all interviews to facilitate language interpretation and translation. Lastly, these findings are also not statistically generalizable, but the conceptual construction of archetypal clusters may exist in other contexts. The study’s conclusions should be interpreted with these limitations in consideration.

## Conclusions

Public and private dementia research funders play a pivotal role in supporting research D&I through diverse dissemination, implementation support and research ecosystem capacity-building activities informed by each sector’s unique resources and value systems. Public funders’ strategies focussed on societal benefits and impact. In contrast, private funders’ strategies centred on delivering value to industry donors and individual contributors, whilst enhancing fair market commercial outcomes. Public and private funders experience persistent challenges with navigating roles and responsibilities amongst the actors within the research ecosystem and steering capacity-building resources to improve implementation outcomes. These findings offer valuable contextual insights to guide the strategic selection of activities that address challenges at each phase of D&I. Future research may focus on designing and developing strategic planning tools that fully support funders in optimizing their D&I impact. Adopting a research ecosystem approach presents a promising pathway to overcome persistent D&I challenges in dementia research and other fields.

## Supplementary Information

**Additional file 1.**

## Abbreviations

**PPP**: Public–private partnerships
**D&I**: Dissemination and implementation
**IKT**: Integrated knowledge translation
**NWO**: Dutch Organization for Scientific Research (Dutch Research Council)
**ZonMw**: The Netherlands Organization for Health Research and Development

## Declarations

### Availability of data and materials

The datasets generated and/or analysed during the current study are not publicly available to preserve the privacy and integrity of participants, but data may be made available from the corresponding author on reasonable request.

## References

[1] Chen S, Cao Z, Nandi A, Counts N, Jiao L, Prettner K, et al. The global macroeconomic burden of Alzheimer’s disease and other dementias: estimates and projections for 152 countries or territories. Lancet Glob Health. 2024;12(9):e1534–43. 39151988 10.1016/S2214-109X(24)00264-X

[2] Netherlands Ministry of Health, Welfare and Sport. (2021). National Dementia Strategy (NDS) 2021–2030.

[3] Anonymous Joint Program -- Neurodegenerative Disease Research and Innovation Strategy. *UK Medical Research Council*.

[4] Annika van de Belt, Janet van den Boer, Albertus Laan, Harm Eskes&nbsp: Evaluatie ZonMw programma Memorabel. *ZonMw*. 2023.

[5] ZonMw&nbsp: Dementia Research Programme 2021–2030.

[6] Pulford J, El Hajj T, Tancred T, Ding Y, Crossman S, Silvester L, et al. How international research consortia can strengthen organisations’ research systems and promote a conducive environment and culture. BMJ Glob Health. 2023;8(4):e011419. 37028811 10.1136/bmjgh-2022-011419 PMC10083781

[7] Morrison M, Mourby M, Gowans H, Coy S, Kaye J. Governance of research consortia: challenges of implementing responsible research and innovation within Europe. Life Sci Soc Policy. 2020;16(1):13. 33190636 10.1186/s40504-020-00109-z PMC7667809

[8] Zhu EM, Buljac-Samardžić M, Ahaus K, Sevdalis N, Huijsman R. Implementation and dissemination of home- and community-based interventions for informal caregivers of people living with dementia: a systematic scoping review. Implement Sci. 2023;18(1):1–60. 37940960 10.1186/s13012-023-01314-y PMC10631024

[9] Lourida I, Abbott RA, Rogers M, Lang IA, Stein K, Kent B, et al. Dissemination and implementation research in dementia care: a systematic scoping review and evidence map. BMC Geriatr. 2017;17(1):147. 28709402 10.1186/s12877-017-0528-y PMC5513053

[10] Stroud C, Larson EB. Meeting the challenge of caring for persons living with dementia and their care partners and caregivers. 1st ed. Washington, D.C: National Academies Press; 2021. 33625814

[11] Warren A. An integrative approach to dementia care. Frontiers in aging. 2023;4:1143408. 36873742 10.3389/fragi.2023.1143408 PMC9978191

[12] Smith JD, Li DH, Rafferty MR. The implementation research logic model: a method for planning, executing, reporting, and synthesizing implementation projects. Implement Sci. 2020;15(1):84–12. 32988389 10.1186/s13012-020-01041-8 PMC7523057

[13] Braithwaite J, Churruca K, Long JC, Ellis LA, Herkes J. When complexity science meets implementation science: a theoretical and empirical analysis of systems change. BMC Med. 2018;16(1):63. 29706132 10.1186/s12916-018-1057-z PMC5925847

[14] Zullig LL, Drake C, Check DK, Brunkert T, Deschodt M, Olson M, et al. Embedding implementation science in the research pipeline. Transl Behav Med. 2024;14(2):73–9. 37688798 10.1093/tbm/ibad050

[15] Leggat FJ, Wadey R, Day MC, Winter S, Sanders P. Bridging the know-do gap using integrated knowledge translation and qualitative inquiry: a narrative review. Qualitative research in sport, exercise and health. 2023;15(2):188–201.

[16] Gagliardi AR, Berta W, Kothari A, Boyko J, Urquhart R. Integrated knowledge translation (IKT) in health care: a scoping review. Implement Sci. 2016;11(38):38. 26988000 10.1186/s13012-016-0399-1 PMC4797171

[17] Triplett NS, Woodard GS, Johnson C, Nguyen JK, AlRasheed R, Song F, et al. Stakeholder engagement to inform evidence-based treatment implementation for children’s mental health: a scoping review. Implement Sci Commun. 2022;3(1):82. 35906675 10.1186/s43058-022-00327-w PMC9338493

[18] Brownson RC, Colditz GA, Proctor EK. Dissemination and implementation research in health: translating science to practice. 1st ed. United Kingdom: Oxford University Press; 2012.

[19] Brantnell A, Baraldi E, van Achterberg T, Winblad U. Research funders’ roles and perceived responsibilities in relation to the implementation of clinical research results: a multiple case study of Swedish research funders. Implement Sci. 2015;10(1):100. 26183210 10.1186/s13012-015-0290-5 PMC4506440

[20] Smits PA, Denis J. How research funding agencies support science integration into policy and practice: an international overview. Implement Sci. 2014;9(1):28. 24565209 10.1186/1748-5908-9-28 PMC3939639

[21] McLean RKD, Graham ID, Tetroe JM, Volmink JA. Translating research into action: an international study of the role of research funders. Health Res Policy Syst. 2018;16(1):44. 29793541 10.1186/s12961-018-0316-y PMC5968540

[22] van der Linden B, Dunham KM, Siegel J, Lazowick E, Bowdery M, Lamont T, et al. Health funders’ dissemination and implementation practices: results from a survey of the Ensuring Value in Research (EViR) Funders’ Forum. Implement Sci Commun. 2022;3(1):36. 35351211 10.1186/s43058-022-00273-7 PMC8966333

[23] Leeman J, Birken SA, Powell BJ, Rohweder C, Shea CM. Beyond “implementation strategies”: classifying the full range of strategies used in implementation science and practice. Implement Sci. 2017;12(1):125. 29100551 10.1186/s13012-017-0657-x PMC5670723

[24] Zurynski Y, Smith CL, Knaggs G, Meulenbroeks I, Braithwaite J. Funding research translation: how we got here and what to do next. Aust N Z J Public Health. 2021;45(5):420–3. 34251704 10.1111/1753-6405.13131

[25] Nilsen P, Bernhardsson S. Context matters in implementation science: a scoping review of determinant frameworks that describe contextual determinants for implementation outcomes. BMC Health Serv Res. 2019;19(1):189. 30909897 10.1186/s12913-019-4015-3 PMC6432749

[26] Chambers DA, Emmons KM. Navigating the field of implementation science towards maturity: challenges and opportunities. Implement Sci. 2024;19(1):26. 38481286 10.1186/s13012-024-01352-0 PMC10936041

[27] Zhu EM, Buljac-Samardžić M, Ahaus K, Huijsman R. Transforming dementia research into practice: a multiple case study of academic research utilization strategies in Dutch Alzheimer Centres. Health Res Policy Syst. 2025. 10.1186/s12961-024-01266-9. 39762851 10.1186/s12961-024-01266-9 PMC11702214

[28] Oortwijn W, Reijmerink W, Bussemaker J. How to strengthen societal impact of research and innovation? Lessons learned from an explanatory research-on-research study on participatory knowledge infrastructures funded by the Netherlands Organization for Health Research and Development. Health Res Policy Syst. 2024;22(1):81–12. 38978042 10.1186/s12961-024-01175-x PMC11229179

[29] Dreves MAE, Harten AC, Visser LNC, Rhodius‐Meester H, Köhler S, Kooistra M, Papma JM, Honey MIJ, Blom MM, Smets EMA, Vugt ME, Teunissen CE, Flier WM. Rationale and design of the ABOARD project (A Personalized Medicine Approach for Alzheimer's Disease). Alzheimer's Dement Transl Res Clin Intervent 2023;9(2):e12401–n/a. 10.1002/trc2.12401 PMC10242186 37287472

[30] Stenfors T, Kajamaa A, Bennett D. How to … assess the quality of qualitative research. Clin Teach. 2020;17(6):596–9. 32790137 10.1111/tct.13242

[31] Timmermans S, Tavory I. Theory construction in qualitative research: from grounded theory to abductive analysis. Sociol Theory. 2012;30(3):167–86.

[32] Creswell JW. Research design: qualitative, quantitative, and mixed methods approaches: 3rd ed. Sage; 2009. 10.7748/nr.12.1.82.s2 28718745

[33] Williams M, Moser T. The art of coding and thematic exploration in qualitative research. Int Manag Rev. 2019;15(1):45–72.

[34] Braun V, Clarke V. Using thematic analysis in psychology. Qual Res Psychol. 2006;3(2):77–101.

[35] Kislov R, Pope C, Martin GP, Wilson PM. Harnessing the power of theorising in implementation science. Implement Sci. 2019;14(1):103. 31823787 10.1186/s13012-019-0957-4 PMC6905028

[36] Moret M, Reuzel R, van der Wilt GJ, Grin J. Validity and reliability of qualitative data analysis: interobserver agreement in reconstructing interpretative frames. Field Methods. 2007;19(1):24–39.

[37] Tong A, Sainsbury P, Craig J. Consolidated criteria for reporting qualitative research (COREQ): a 32-item checklist for interviews and focus groups. Int J Qual Health Care. 2007;19(6):349–57. 17872937 10.1093/intqhc/mzm042

[38] Jull J, Giles A, Graham ID. Community-based participatory research and integrated knowledge translation: advancing the co-creation of knowledge. Implement Sci. 2017;12(1):150. 29258551 10.1186/s13012-017-0696-3 PMC5735911

[39] Adner R. Match your innovation strategy to your innovation ecosystem. Harvard Bus Rev. 2006;84(4):98–107. 16579417

[40] Adner R. Ecosystem as structure. J Manage. 2017;43(1):39–58.

[41] Talmar M, Walrave B, Podoynitsyna KS, Holmström J, Romme AGL. Mapping, analyzing and designing innovation ecosystems: the ecosystem pie model. Long Range Plann. 2020;53(4):101850.

[42] Noordegraaf M. Hybrid professionalism and beyond: (new) forms of public professionalism in changing organizational and societal contexts. J Prof Organ. 2015;2(2):187–206.

[43] Noordegraaf M, Brock DM. Protective and connective professionalism: what we have learned and what we still would like to learn. J Prof Organ. 2021;8(2):228–36.

[44] Pérez Jolles M, Willging CE, Stadnick NA, Crable EL, Lengnick-Hall R, Hawkins J, et al. Understanding implementation research collaborations from a co-creation lens: recommendations for a path forward. Front Health Serv. 2022. 10.3389/frhs.2022.942658. 36908715 10.3389/frhs.2022.942658 PMC10003830

[45] Bryson JM, Crosby BC, Stone MM. Designing and implementing cross-sector collaborations: needed and challenging. Public Adm Rev. 2015;75(5):647–63.

[46] Metz A, Albers B, Burke K, Bartley L, Louison L, Ward C, et al. Implementation practice in human service systems: understanding the principles and competencies of professionals who support implementation. Adm Soc Work. 2021;45(3):238–59.

[47] Moore JE, Rashid S, Park JS, Khan S, Straus SE. Longitudinal evaluation of a course to build core competencies in implementation practice. Implement Sci. 2018;13(1):106. 30081921 10.1186/s13012-018-0800-3 PMC6080520

[48] Goense PB, Wilschut M, Fleuren MAH, Stals K, Goossens F, Boendermaker L. De ontwikkeling van implementatienetwerken. 2018.

[49] Brownson RC, Cabassa LJ, Drake BF, Shelton RC. Closing the gap: advancing implementation science through training and capacity building. Implement Sci. 2024;19(1):46–54. 38961482 10.1186/s13012-024-01371-x PMC11223366

[50] De Geest SM, Akre C, Aubert CE, Brauchli P, Brunkert T, Dhaini S, et al. Accelerating innovation: implementation science as a cornerstone of high-performance Swiss research infrastructures. Swiss Med Wkly. 2025;155(10):4501. 41100820 10.57187/s.4501

[51] Villani E, Greco L, Phillips N. Understanding value creation in public-private partnerships: a comparative case study. J Manage Stud. 2017;54(6):876–905.

[52] Spaapen J, van Drooge L. Introducing “productive interactions” in social impact assessment. Res Eval. 2011;20(3):211–8.

[53] Van Der Wal Z, De Graaf G, Lasthuizen K. What’s valued most? Similarities and differences between the organizational values of the public and private sector. Public Adm. 2008;86(2):465–82.

[54] Liu LX, Clegg S, Pollack J. The effect of public-private partnerships on innovation in infrastructure delivery. Proj Manag J. 2024;55(1):31–49.

[55] Fric U, Lutman T, Mlinar T. State aid in academia-industry cooperation: an overview of the existing conditions and challenges through the ExSACT Project. CERN ideaSquare J Exp Innov 2024;8(2).

[56] Stevens H, Huys I. Intellectual property in early-phase research public–private partnerships in the biomedical sector. In: The Cambridge Handbook of Public–Private Partnerships, Intellectual Property Governance, and Sustainable Development. Cambridge University Press; 2018:109–140.

[57] Pujotomo D, Syed Hassan SAH, Ma’aram A, Sutopo W. University–industry collaboration in the technology development and technology commercialization stage: a systematic literature review. J Appl Res High Educ. 2023;15(5):1276–306.

[58] Oh AY, Emmons KM, Brownson RC, Glasgow RE, Foley KL, Lewis CC, et al. Speeding implementation in cancer: the National Cancer Institute’s Implementation Science Centers in Cancer Control. JNCI J Natl Cancer Inst. 2023;115(2):131–8. 36315080 10.1093/jnci/djac198 PMC9905952

[59] Davis R, D’Lima D. Building capacity in dissemination and implementation science: a systematic review of the academic literature on teaching and training initiatives. Implement Sci. 2020;15(1):1–97. 33126909 10.1186/s13012-020-01051-6 PMC7597006

[60] Tabak RG, Padek MM, Kerner JF, Stange KC, Proctor EK, Dobbins MJ, Colditz GA, Chambers DA, Brownson RC. Dissemination and implementation science training needs: insights from practitioners and researchers. Am J Prevent Med. 2017;52(3):S322-9. 10.1016/j.amepre.2016.10.005 PMC5321656 28215389

