# Supplementary material for: Bridging research and practice for dementia care: strategies and challenges of public and private funders in the dissemination and implementation of dementia research
Source: Health Res Policy Syst. 2026 Jan 14;24:14. doi: 10.1186/s12961-025-01440-7 (PMC12888492; doi:10.1186/s12961-025-01440-7)
Supplement: Supplementary file 1 — Additional file 1. [file 12961_2025_1440_MOESM1_ESM.docx]

Additional file 1. COREQ reporting checklist_Coding framework_Data and interview guide

| Table 1. Data Collection tool (Interview guide) |
| --- |
| Relating to Aim 1: What main activities and strategies were performed by public and private funders of dementia care research to facilitate research dissemination and implementation? |
| 1. [Release of research/Dissemination] How do funders enhance research output usability for the general public? (e.g., simplified language, establishing information hub/repository, press briefings, media exchange?) 2. [Knowledge exchange activities] What are activities (or infrastructures) that funders conduct (or utilize) to bring inter-agency stakeholders together to share knowledge and act upon research findings to create societal impact? 3. [Implementation] Are there formal funding schemes (e.g., financing instruments) to support awardees (research or other) to undertake implementation projects (e.g., clinical guideline implementation projects)?    1. What implementation requirements are explicit in grants?    2. What are implementation steps required by applicants?    3. How are they expected to be reported to funders?    4. How do you monitor these requirements? 4. [Building capacity/infrastructure] Are there specific awards or funding schemes that support building capacity for dissemination or implementation activities? 5. [Implementation research] How do funders provide support for implementation science research (e.g., determining and comparing implementation strategy effectiveness) |
| Relating to Aim 2: What related organizational and external challenges did public and private funders encounter in facilitating research dissemination and implementation? |
| 1. What challenges do funding agencies experience when engaging external organizations, such as resistance from healthcare providers and other implementing agencies, to create research impact? 2. What infrastructure or mechanisms are in place to facilitate co-financing of healthcare research initiatives, and what challenges arise in this process? 3. What challenges do funders have to disseminate research knowledge? How would you like to receive the research knowledge (results) so it is more appropriate to share? 4. How do government policies affect the implementation and support of healthcare research initiatives funded by academic research funders? 5. What external environmental factors, such as healthcare networks and industry trends, impact the funding and execution of healthcare research projects? |

| Table 2. Coding framework | | | |
| --- | --- | --- | --- |
| (inductive) Strategy clusters and corresponding [original] practice areas | Second order codes [Selective code]  (Funder’s strategies) | First order codes  [Axial codes]  (Funder’s activities) | Quotes |
| Dissemination (release of findings, dissemination) | (indirect) Provide incentives | Provide financial prizes | *We have the (PhD thesis prize). They [PhD candidates] have to send in their summary. But then in easy language so everyone can understand it. So that's really for the communication. And we also have research talent award, but that's not really for communication for their research. But I think the thesis prize is especially for communication. (Private; respondent 12)* |
|  | (*indirect*) Mandate action | Require publications following FAIR data principles | *We also budgeted quite some money for FAIR data. We have spent much money, because we want to make sure that projects have organized that correctly. Within the consortia we ask for data managers. We will also be focusing a lot on this in the near future. It is a part of [consortium] to give attention to reusing data, the open access of data. (Public; respondent 2)* |
|  |  | Require open access publications | I*t's a requirement for publications to do Open Access, so should always be Open Access, and you're also obliged to share your results or data with people that do a reasonable request (Public; respondent 5)* |
|  | (indirect) Provide guidance | Research output guided by responsible research practices | *We have some tools and frameworks, that we use to enhance the impact of projects. It consists of two things. One of them is BVO: Bevorderen Verantwoorden Onderzoekspraktijken. That is like really… in the criteria we ask a lot of things. So at the beginning of a project, to make sure that they're already looking into things. (public; respondent 1)* |
|  |  | Researchers are advised to create and provide (dissemination) impact plan | *Researchers have to complete an application form, which is specifically built according that impact plan approach. So every researcher, component or construction has to complete in that registration, that application form, fills in parts of that impact plan approach, basically. With, of course, the advice on our side, really start with the end in mind. And then, reason back. (public; respondent 8)*  *We had a big part in dissemination of the results, putting on some nice texts on our websites or LinkedIn, funding certain parts, but now we try to be more proactive (…) we want to say to them, “Hey, this looks interesting. Did you already think about certain implementation stages?” So we try and steer more active. (Private; respondent 17)* |
|  | (direct) Translate academic jargon | Translating research findings to share with donors | *I received the [mid-term and end] reports of the researchers. And then decide whether it's interesting for our target audience. Our main target audience is the informal caregivers and the family. So what does this research mean for them? What are some practical tips that I can take from the research? And for more fundamental research that's difficult. I also use those studies because we can show what we do with their money because they're also donors, so they give their money and then we want to show what we do with the money. (Private; respondent 12)* |
|  |  | Send ‘translated’ research to wider audiences through targeted newsletters | *We use the newsletter of the elderly department. So that is our main channel for the newsletter. We have the LinkedIn page. We have the website, of course. We have some articles that we publish on the website. But then, we also bring them further through LinkedIn. (Public; respondent 1)* |
|  | (direct) Develop decision support tools (evidence synthesis) for policymakers | Develop evidence synthesis to set research agenda | *We need those [evidence] synthesis as soon as possible to basically show the world that this is what came out. This is what still is lacking on different angles, like process, but also [valorisation]. And what can be done in the next period? Especially the health care environment, relating to all those accords. (Public; respondent 8)* |
|  |  | Support policymakers by providing input (evidence) | *On the national level, we monitor the agenda of Parliament. With input for discussions in the Parliament. And questions to ask or motions to make. (…) We also have some colleagues who participate in the development of professional guidelines and guidelines in the Netherlands are the tool to implement proven interventions. (Private; respondent 14)* |
|  | (direct) Adapt evidence into multi-modal formats | Share through media to reach caregivers and end-users | *If it's very big news, then we want to reach to the media for a bigger audience. But also, for example, in the big consortium, I also do the communication there and then I use the different partners to reach different target audience, if it's more for caregivers or for another target audience that [the funder] does not reach. (Private; respondent 12)* |
|  |  | Engage policymakers to share through TV (media) | *What we do is we try to collaborate with researchers to inform Members of Parliament and politicians. Not very often, but we do. And of course we invite them to the media to tell their stories about how they do things and what they what about the results. We have a TV show with researchers telling about what they are doing and why it's important for people with dementia (…) we like those researchers who can talk about what they are doing and their results in a way “normal” people can understand (Private; respondent 13)* |
|  |  | Share research output though small videos/movies | *We are also making small movies about the projects that we fund already, to show other municipalities what they are doing and what is helping them, what the results are, to inspire them, to make them learn from each other already before the learning community starts. (Public; respondent 3)* |
|  | Challenges to dissemination | Low engagement and reach of public audiences | *For communication, the problem is that you cannot always reach the people that you want to reach, but you reach the people that want to read it. I think that's a really big issue. How do you reach the people that really need to know it? And I think that's in general challenging. But there are just hard to reach target audience and especially for older people…maybe not that used to the Internet. How do you reach them? (Private; respondent 12)*  *I want to develop new strategies to connect researchers and implementers. Because people doing research only think about articles and presentations. But this is not enough… because people working in dementia care, they don't go to those presentations. They don't read those articles. I think those consortia researchers even have to change their ways of doing research. Participation of people with dementia, carers, health carers, welfare carers... they have to participate along. So we have to think of other ways of doing research. (Private; respondent 14)* |
| Implementation support (implementation) | (indirect) Provide incentives | Financial subsidies: VIMPS, PEARLS, Implementation vouchers | *We have specific calls for proposal. So, the VIMP, dissemination and implementation grant is €50,000 for finished ZonMw projects. (public; respondent 1)*  *We have vouchers that you can actually bring in within implementation experts within your project. (public; respondent 7)* |
|  | (indirect) Mandate action | Require implementation support specialist in (consortium) research project team | *We have the implementation specialists that can also give advice on how to work on the implementation plan, and what we also advise projects to do is find their own capacity expertise in their university, or another place…so an implementation specialist for themselves, integrated in the project team. (public; respondent 5)* |
|  |  | Require impact plan from consortium projects | *We asked all the consortia to write an impact plan. So the impact plan is based on dissemination, how you want to do that, or communications. So they had to make a plan. This plan is changeable. So dynamic with time. They can learn from it. They can change it during the next years. (public; respondent 1)*  *So every researcher, component or construction has to complete in that registration, that application form, fills in parts of that impact plan approach, basically. With, of course, the advice on our side, really start with the end in mind. And then, reason back. (Public; respondent 8)*  *Now we try to establish more conditions. We want to partake in your consortium, if you covered these and these kinds of conditions that we actually want. So we want to certain level of patient participation, we want you to really focus on the end user and user community, for example. We want you to formulate, for example, impact routes. So what are between your primary and secondary outcomes? What are actually your goals? If it comes to what are your results going to mean for the society? What is it going to mean for the patients in the end. So we want them to focus on all these kinds of different aspects. (private; respondent 17)* |
|  | (indirect) Provide guidance | Provide guidance to develop a theory of change (impact model) | *An instrument that we like to use is based on the theory of change and the effectenkaart [effect cards], which are a way of making a theory of change or a change path, an impact model. (Private; respondent 13)* |
|  |  | Guide implementation stages at early stages of project planning | *We had a big part in dissemination of the results, putting on some nice texts on our websites or LinkedIn, funding certain parts, but now we try to be more proactive (…) we want to say to them, “Hey, this looks interesting. Did you already think about certain implementation stages?” So we try and steer more actively. (Private; respondent 17)* |
|  | (direct) Broker partnerships | Stimulate (shared) research ownership,  connecting with research ecosystem stakeholders, making usable products for societal benefit, and developing effective dissemination and implementation strategies. | *we asked first the practice to come up with solutions or problems that they encounter doing their jobs, and then ask researchers to make a plan how to solve the problem with research. So then we actually did it the other way around, and within the application we asked them…it is based on a on a practical problem or a practical solution already, so that is a part in which they have to mention how they will implement that or incorporate that within the current situation. So that's something that we need to ask. And we have for this grant cycle an advisory board, which is more on the societal impact side, not only with people with mention their carers, but also with people working in practice or several kinds of backgrounds. (Private; respondent 13)*  *We have also trained our colleagues to be facilitated to do those big matchmaking and collaboration sessions. So, yes. We are very much in how we can help [funder] give the information, but also help with getting partners to collaborate with each other. (Public; respondent 9)* |
|  | (direct) Provide valorization support | Build networks among funders and implementation experts to bridge knowledge gaps | *We started with founding the Dutch implementation collaboration. So it's broader than only valorization centers, but in the specific corner of our organization that focuses on fundamental research and E health. And so those domains of research. They talk more about valorization instead of implementation and they have strong networks also with [other funders]* (Public; respondent 5) |
|  |  | Use valorization structures, including incubators, accelerators (e.g., Health Impact Accelerator), and technology transfer offices, to advance dementia research accessibility and commercialization | ***We try to see how we can improve that equal collaboration, even when it's a small company and a bigger university. But it's also nice to see that a lot of parties go for second or first collaborations with partners. An example that we use for [consortium] is that [health innovation accelerator], they've worked together with the research group before ABOARD, and that's how they got connected. And then now they are involved in this huge national consortium. It's good to see that when people find each other, they also keep working together. (private; respondent 19)*** |
|  |  | Maintain relationship with insurance and regulatory agencies | Engaging with insurance companies for implementation and de-implementation - *Any new innovation has the backside that the old stuff should be stopped. And does that go automatically? Mostly not. Especially when the new thing is not being reimbursed and the old thing keeps, and it is the old practice. There are specific programs, choosing wisely, lists of things… we help along the development of that. (*Public; respondent )  Regulatory parties (guidelines) - *If we know that there is a party that is responsible, for example, the guidelines for medical specialists. Then it would be a requirement that you turn over your results or discuss your results with the guideline party at the Federation of Medical Specialists. We know if these results give these results that the guideline has to be changed. And then there are also agreements with the Federation of Medical Specialists that they will be available for party to take this up. And that's from an implementation side of part is this very effective. (*Public; respondent 5)  *You cannot only define impact as words. This is how we do it as a definition. You create impact together. That is my vision about impact. That is what I see here. It is important when you collaborate with others, public or private parties, the responsibility that you have with these others, the walk the path of impact, that is where you need to invest in. Then you invest together in the front, with those parties. Those parties know that it is their responsibility, to gain the impact that we want to have. I think that it is the only way that how to get to impact, can work. Not by a definition or something else. (*Public; respondent 9) |
|  |  | Securing sustainable financing, enhancing business models, and creating pathways for scaling dementia research | *I'm positive in the sense that it opens up the eyes of researchers to look more in the future. Not just specifically in their own scientific fields but actually really think hey what can we do with all these kinds of results. And then so you don't need always valorization, but it can also actually lead to new research or the correct next steps. (…) And if there comes a start up out of it or a spin off, that's great. We also try to focus primarily on that the end results come to the patients in a in an honest way, of course. And to a right price market for example, that's very important for us. (Private; respondent 17)*  *So we're going to form a panel of external investors and they are also going to rate the project on their market viability (…) there's also the idea or the goal to also start a phase three so that projects that come from the phase two projects can also apply for future follow up funding. So we really try to guide the projects and the innovation from the start from different levels, phase one or phase two, and then can go on to the to the next funding possibility. (Private; respondent 19)* |
|  | Challenges to implementation support | Unclear research D&I roles among public funders, private funders, and research teams led to conflicting expectations | *We try to stimulate the researchers. We are funding research. So, we try to stimulate the implementation. ‬‬(…) But the field will have to take it on. Also, if you want it to work for the coming years, there has to be some money [invested] from the organization itself, because otherwise, after one year of money from [funder], then who is going to pay for it then? They have to look at how they are going to pay for this. We can do things to stimulate it or to give the first push, but the field has to take it…‬ (Public; respondent 1)*  *What I do find, is that most of the times, researchers don't see the implementation as their task. I know that they are right from a certain point of view. But if they are thinking like that from the beginning, that implementation is not their path, and they are not involving the partners who will be responsible for the implementation, I think they will not be there where we want to be. So, I think that perception, that implementation is not our task to do that, but I think that as a researcher, because we are doing research to improve the field coma to improve the practice, that I think that we should as researchers, think about it. Who should we involve in our research to make sure that my research will be implemented? (Public; respondent 10)* |
|  |  | Researchers did not adequately engage with these interdisciplinary expertise and resources to support research D&I | *There's a pathway from the side, from the bench to bedside. And I think you cannot expect that the researchers at the bench bring it to the bed. It's a chain and we think it's important that you talk to the right person, and that they think forward, to whom I can hand it over. Most of the time, what happens is [that] they did something at the bench. They discovered it. And then they started over again with another project. (Respondent 16; Private funder)* |
| Research ecosystem capacity building (knowledge exchange and partnering, building capacity and infrastructure, implementation research) | Invest in implementation workforce competencies | Evolving funder’s internal (individual and team) competencies | *It is interesting to note that on the other team, the Research and Society Team, there are three people working on public patient involved. And two people working on communication, scientific communication, and as a whole then we cover the whole spectrum of funding, but also checking on relevance, advising researchers on how to involve people with dementia, communicating about the results. So I think we need both teams to really make more impacts (private; respondent 13)*  *We also have some colleagues who participate in the development of professional guidelines and guidelines in the Netherlands are the tool to implement proven interventions (private; respondent 14)*  *If you are bilingual like speaking the language of the researcher as well as of the practice, then that will help in building that bridge between the practice and science. But you need to understand both sides of the bridge, and I hope that those impact managers can get the trust of both parties to be able to build that bridge and to walk over that bridge and maybe have more responsibility in that. (private; respondent 13)* |
|  |  | Develop external workforce (human resources) | *And they are financing 10 implementation science practitioners. It is for fellows. They get an education implementation science. So, they use it for their own field of interest. (public; respondent 1)*  *We noticed that it is not enough to ask certain questions on implementation. We also need to build the infrastructure… help to build the infrastructure of people and networks that is needed to do that implementation. (…) We are becoming a little bit stricter when it comes to implementation. But we also noticed that we need to help to fill that knowledge. (public; respondent 7)* |
|  | Strengthen professional collaborative networks | Engaging networks of partnering organizations | *We tried to reach the general public more on social media. But I think there are other partners that can do that better. And also with caregivers, for example. Zorgvoorbeter (https://www.zorgvoorbeter.nl) , the NKN, and the Dutch Memory clinic. We can use those partners to reach the caregivers. (Private; respondent 12)* |
|  |  | Build public-private partnerships | *we can invest that in public private partnerships. So we said, OK, so we're going to bring our knowledge and our networks together. And so we're going to focus on these different disease areas and we want to have more longer research lines. For example, over 10 years. (Private; respondent 17)* |
|  |  | Build collaborative infrastructures (knowledge planes, learning networks) | *We have different types of infrastructures that we also finance, and we also actually see that as a part of our implementation policy (…) We also did an evaluation on the different types of collaboratives or infrastructures that we subsidized to see what are the most effective parts of these collaborations. (…) So we have learning networks. We have academic working places. We have knowledge planes. So we have certain types, and last year we gathered twenty types of larger infrastructures that we have been financing and we did an evaluation. (Public; respondent 7)* |
|  | Build research governance structures | Public-private research consortium models (shared ownership, risk, responsibilities) | *[Funder] is kind of following that trend, to use it as a means to see what can be done with the results. Are we even achieving those results? Is it deliverable? So, the output of a project. Then, the next step: outcome. What can stakeholders do with that? And to really guide the consortium in achieving those outcomes, we have so-called productive interactions. (public; respondent 8)*  *We have also trained our colleagues to be facilitated to do those big matchmaking and collaboration sessions. We are very much interested in how we can help [funder] give the information, but also help with getting partners to collaborate with each other. (Respondent 9; Public funder)* |
|  |  | Implementation monitoring and evaluation (metrics, tools, and M&E structures) | *We are now developing the process to find out what we want to monitor. Because when you want to monitor, they call it indicators. We need to know the indicators on how to monitor. We are now in this process, looking at the program, looking at the project, and then, try to find the key indicators that can tell us something about the whole program. (Public; respondent 8)* |
|  | Challenges to research ecosystem capacity building | Low maturity of current D&I infrastructure (unable to monitor and evaluate implementation progress and impact) | *It takes a lot of attention or time to start this program in a good way, and then we have too little time or attention for really following up [on] the results and making sure that it reaches its impact. So that's one of the challenges. And the other thing is, that we do a lot of projects. So it's very difficult to see all these connections between this problem, and then there are a lot of connections. (Public; respondent 5)*  *It is a challenge because we want to be able to say “this is the impact of our project”. But the things that come out of projects that are measurable are the amount of patents and amount of publications. So we have a whole list where they need to fill in. “What are the outputs of your research?” But it is quite hard to measure the societal impact and also the economic impact. So they need to address this in their application. But there's not yet a real strong way for us to assess all the impacts from our project and it is something that we try to develop and make it better. But we have some numbers, for example, on publications. But we all know that it's not the best indicator. (Respondent 19; Private funder)*  *We are now developing the process to find out what we want to monitor. Because when you want to monitor, they call it indicators. We need to know the indicators on how to monitor. We are now in this process, looking at the program, looking at the project, and then, try to find the key indicators that can tell us something about the whole program. (Respondent 8; Public funder)* |
|  |  | Difficult navigating strategic stakeholder engagement in governing research co-funding and public-private research partnerships | *But this is the key of what is happening also because sophisticated research and results and interventions are more quickly highly rated… and people who develop more simple interventions which people with dementia like, they're not for The Lancet or for high rated journals. This is a problem. (Respondent 14; Private funder)*  *I know that one of the problems, for example, for medical products is that it's under European legislation and you have to have a certain certification, which also accounts for very small interventions like an app. And this whole trajectory also involves legal people from Brussel, which counts € 800 per hour for that advice. And the talk about the cost of such territory is about € 150,000 or something. So then they're not able to get this accreditation. So they're not allowed to go to the market. So that's one of the things that I know that are really blocking the implementation or the use of this knowledge. (Respondent 5; Public funder)*  *If a party will generate money from it, then we have a problem with the public money that comes from the Ministry of Health. (…) We have these strict regulations about that you cannot earn money with it and have an advantage over other organizations in the field.‬ (…) we give money to one organization, and they will have an advance on the market and the other organization not… Then, it is stated that it is prohibited. (Respondent 1; Public funder)* |
|  |  | Conflicting value systems for public-private partnerships | *One of the questions that we were asked, was how the program contributes to the national dementia strategy. Specifically on the [goal that] 80% [of people with dementia] has access to meaningful activities etc. We said that we can't do that. There is no direct link between what we're trying to achieve in the field and the access [to research impact] that people have. (…) We can try to colour them with stories from the project [results] and the municipalities with their experience within the program. (Respondent 4; Public funder)* |

| **Table 3. Consolidated criteria for reporting qualitative studies (COREQ): 32-item checklist** | | |
| --- | --- | --- |
| **No. Item** | **Guide questions/description** | **Reported on Page #** |
| **Domain 1: Research team and reﬂexivity** | | |
| *Personal Characteristics* | | |
| 1. Interviewer/facilitator | Which author/s conducted the interview or focus group? | 7 |
| 2. Credentials | What were the researcher’s credentials? E.g. PhD, MD | 7 |
| 3. Occupation | What was their occupation at the time of the study? | 7 |
| 4. Gender | Was the researcher male or female? | n/a |
| 5. Experience and training | What experience or training did the researcher have? | 7 |
| *Relationship with participants* | | |
| 6. Relationship established | Was a relationship established prior to study commencement? | **7** |
| 7. Participant knowledge of the interviewer | What did the participants know about the researcher? e.g. personal goals, reasons for doing the research | 7 |
| 8. Interviewer characteristics | What characteristics were reported about the inter viewer/facilitator? e.g. Bias, assumptions, reasons and interests in the research topic | 7 |

| **Domain 2: study design** | | |
| --- | --- | --- |
| *Theoretical framework* | | |
| 9. Methodological orientation and Theory | What methodological orientation was stated to underpin the study? e.g. grounded theory, discourse analysis, ethnography, phenomenology, content analysis | 8 |
| *Participant selection* | | |
| 10. Sampling | How were participants selected? e.g. purposive, convenience, consecutive, snowball | 7 |
| 11. Method of approach | How were participants approached? e.g. face-to-face, telephone, mail, email | 7 |
| 12. Sample size | How many participants were in the study? | 9 |
| 13. Non-participation | How many people refused to participate or dropped out? Reasons? | 7 |
| *Setting* | | |
| 14. Setting of data collection | Where was the data collected? e.g. home, clinic, workplace | 7 |
| 15. Presence of non-participants | Was anyone else present besides the participants and researchers? | n/a (not applicable) |
| 16. Description of sample | What are the important characteristics of the sample? e.g. demographic data, date | 7 |
| *Data collection* | | |
| 17. Interview guide | Were questions, prompts, guides provided by the authors? Was it pilot tested? | 7 |
| 18. Repeat interviews | Were repeat inter views carried out? If yes, how many? | **7** |
| 19. Audio/visual recording | Did the research use audio or visual recording to collect the data? | 7 |
| 20. Field notes | Were ﬁeld notes made during and/or after the inter view or focus group? | n/a (not applicable) |
| 21. Duration | What was the duration of the inter views or focus group? | 7 |
| 22. Data saturation | Was data saturation discussed? | 7 |
| 23. Transcripts returned | Were transcripts returned to participants for comment and/or correction? | 7 |
| **Domain 3: analysis and ﬁndings** | | |
| *Data analysis* | | |
| 24. Number of data coders | How many data coders coded the data? | 8 |
| 25. Description of the coding tree | Did authors provide a description of the coding tree? | 8 |
| 26. Derivation of themes | Were themes identiﬁed in advance or derived from the data? | 8 |
| 27. Software | What software, if applicable, was used to manage the data? | **8** |
| 28. Participant checking | Did participants provide feedback on the ﬁndings? | **7** |
| *Reporting* | | |
| 29. Quotations presented | Were participant quotations presented to illustrate the themes/ﬁndings? Was each quotation identiﬁed? e.g. participant number | 11-21 |
| 30. Data and ﬁndings consistent | Was there consistency between the data presented and the ﬁndings? | **11-21** |
| 31. Clarity of major themes | Were major themes clearly presented in the ﬁndings? | **11-21** |
| 32. Clarity of minor themes | Is there a description of diverse cases or discussion of minor themes? | **11-21** |
